# Insomnia and depression among bariatric surgery patients: the chain mediating effect of resilience and anxiety

**DOI:** 10.3389/fpsyt.2025.1554239

**Published:** 2025-05-30

**Authors:** Jijian Si, Yixin Zhang, Manyuan Li, Tour Liu

**Affiliations:** ^1^ Department of Clinical Psychology, Tianjin Medical University Central Hospital, Tianjin, China; ^2^ School of Psychology, Shaanxi Normal University, Xi’an, China; ^3^ Faculty of Psychology, Tianjin Normal University, Tianjin, China; ^4^ Key Research Base of Humanities and Social Sciences of the Ministry of Education, Academy of Psychology and Behavior, Tianjin Normal University, Tianjin, China; ^5^ Tianjin Social Science Laboratory of Students’ Mental Development and Learning, Tianjin, China

**Keywords:** bariatric surgery, insomnia, depression, resilience, anxiety

## Abstract

**Objective:**

This study aimed to examine the psychological mechanisms of depression in both bariatric surgery candidates and post-bariatric surgery patients and to explore the roles of resilience and anxiety in these relationships.

**Methods:**

A total of 431 candidates for bariatric surgery were recruited from a hospital. One month after the bariatric surgery, 228 patients continued to participate in the study. All participants completed the Insomnia Severity Index (ISI), Connor and Davidson Resilience Scale (CD-RISC), Generalized Anxiety Disorder 7-item scale (GAD-7), and Patient Health Questionnaire Depression Scale (PHQ-9). A mediation model analysis was used to investigate the mediating role of resilience, the mediating role of anxiety, and the chain mediating role of resilience and anxiety in the relationship between insomnia and depression.

**Results:**

Insomnia positively correlated with depression through the mediating role of resilience, the mediating role of anxiety, and the serial mediating effect of resilience and anxiety among bariatric surgery candidates. However, only a direct effect of insomnia on depression and the mediating role of anxiety were found in post-bariatric surgery patients.

**Conclusion:**

The findings demonstrated that insomnia leads to an increase in depression among bariatric surgery patients, with resilience and anxiety playing significant mediating roles. This also highlights the need for targeted resilience-enhancing interventions in obese patients who are about to undergo bariatric surgery.

## 1 Introduction

Obesity has become a growing public health concern in China, with its prevalence among adults rising significantly from 3.1% in 2004 to 8.1% in 2018 (1). Given that bariatric surgery is an effective means of weight loss, patients with severe obesity tend to undergo bariatric surgery (2). Notably, bariatric surgery candidates are vulnerable to mental health impairments, with more than 36% of bariatric surgery candidates being diagnosed with depression (3). However, few studies have explored the psychological mechanisms underlying depression in bariatric surgery candidates (4).

In addition to experiencing depressive symptoms, bariatric surgery candidates also struggle with insomnia (5). They consistently report poorer sleep quality compared to the general population controls, characterized by prolonged sleep onset latency, reduced total sleep time, and diminished sleep efficiency (6). These sleep-related problems have been shown to significantly impact mental health, including depression (7, 8). Therefore, we infer that insomnia is positively related to depression among candidates for bariatric surgery.

Resilience is the ability to protect individuals from adversities and stress (9). When confronted with stressors, resilience serves a protective function by preserving mental health (10). Previous research has also observed that individuals with higher resilience are less likely to experience depressive symptoms (11). However, insomnia, a common source of daily stress, appears to undermine resilience (12). Specifically, insufficient sleep duration and poor sleep quality are associated with lower resilience levels (13). Moreover, previous studies have shown that resilience mediates the relationship between stressors and depression (14, 15). Thus, we propose that resilience plays a mediating role in the relationship between insomnia and depression among candidates for bariatric surgery.

Anxiety might also mediate the relationship between insomnia and depression. Insomnia has been identified as a risk factor for anxiety (16). For instance, studies on sleep deprivation have demonstrated that sleep loss increases level of anxiety (17). Anxiety and depression are commonly comorbid (18), and anxiety often serves as a precursor to depression (19). Horn and Wuyek (20) reviewed prior research and concluded that various forms of anxiety, including social anxiety, generalized anxiety disorder, separation anxiety disorder, and specific phobias, are associated with an elevated risk of subsequent depression. Hence, we propose that anxiety plays a mediating role in the relationship between insomnia and depression among candidates for bariatric surgery.

As mentioned previously, resilience enables individuals to quickly recover from stressful events, reducing their impact on mental health (9). When individuals suffer from insomnia, resilience plays a protective role and reduces the anxiety experienced by insomnia (21, 22). Anxiety, in turn, is associated with an increased risk of depressive symptoms (23).Hence, we believe that resilience and anxiety play a chain-mediating role in the relationship between insomnia and depression among candidates for bariatric surgery.

**Figure 1:**
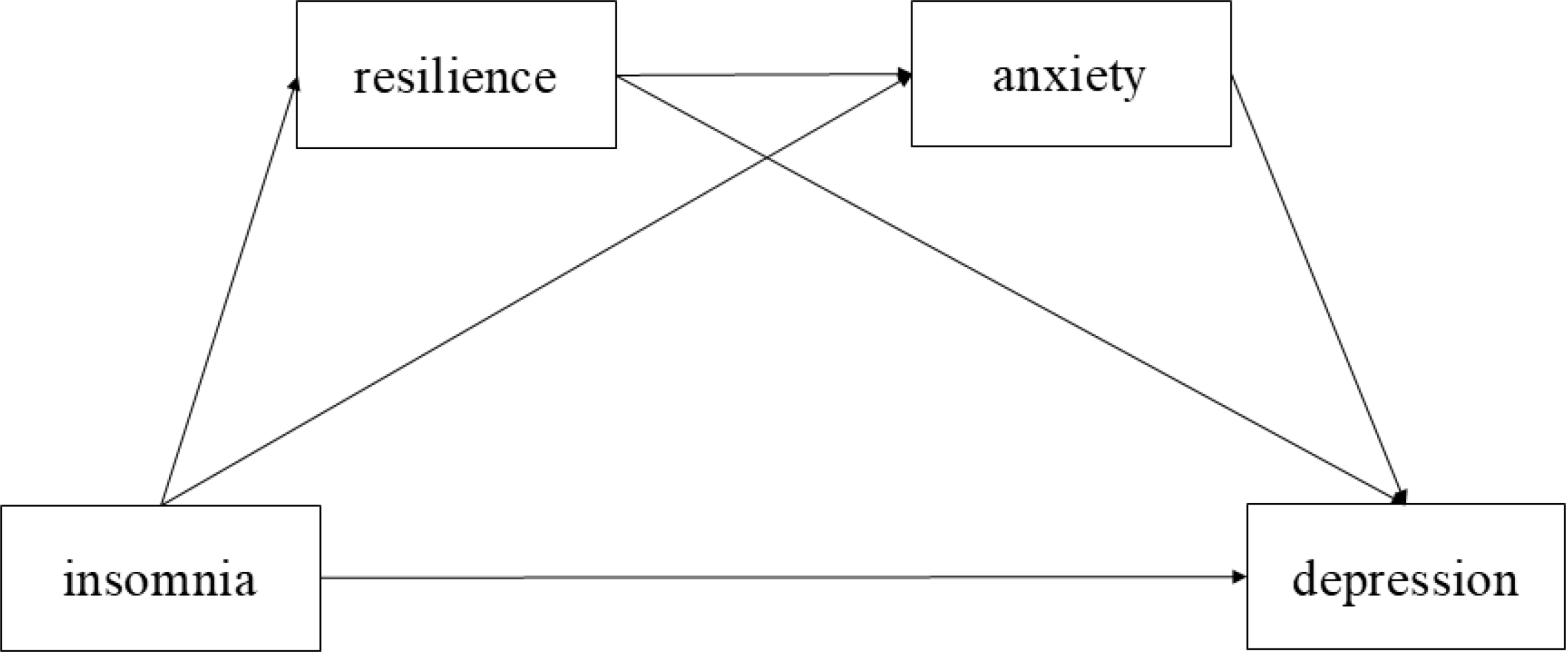
The hypothetical model of the relationships of insomnia, resilience, anxiety, and depression.

Overall, the present study developed a hypothetical model of the relationship between insomnia and depression among candidates for bariatric surgery, in which resilience and anxiety act as mediators (**Figure 1**). In addition, bariatric surgery has been shown to significantly improve sleep quality among obese individuals (24). However, Riemann et al. (25) suggested that insomnia may impair individuals’ emotional regulation capacity, thereby contributing to the development of depression. This may indicate that the hypothetical model is robust among post-bariatric surgery patients.

## 2 Methods

### 2.1 Participants

From May 2020 to August 2022, a total of 431 bariatric surgery candidates (Mean age = 31.80, SD = 7.34) were recruited from a hospital in Tianjin, China. One month after completing bariatric surgery, 228 patients continued to participate (Mean age = 31.05, SD = 7.12). They completed psychological health assessments, including evaluations of insomnia, resilience, anxiety, and depression, both before and one month after the surgery.

Potential bariatric surgery candidates were selected based on the guidelines of the Chinese Clinical Guidelines for the Surgery of Obesity and Metabolic Disorders (2024 edition), which strongly recommend surgery for patients with a BMI ≥32.5 and for those with a BMI between 27.5 and 32.5 who have comorbidities such as type 2 diabetes mellitus or gastroesophageal reflux disease. Additional eligibility criteria are detailed in the guidelines (26).

### 2.2 Measurements

#### 2.2.1 Insomnia

The 7-item scale Insomnia Severity Index (ISI) was used to assess the insomnia of participants (27). The first three items assess difficulty falling asleep, staying asleep, and early morning awakenings, respectively. The remaining four items measured sleep satisfaction, interference with daily functioning, noticeability of sleep problems by others, and distress caused by sleep problems. Each item is rated on a 5-point scale. A higher total score implied a higher degree of insomnia. Cronbach’s *α* coefficient of the ISI for both bariatric surgery candidates and post-bariatric surgery patients was 0.91.

#### 2.2.2 Resilience

Resilience was measured using the Chinese version of the Connor and Davidson Resilience Scale (CD-RISC), which consists of 25 items (28). The CD-RISC was designed to evaluate an individual’s ability to protect themselves from stress and adversity (e.g., “I feel in control of my life”). Responses were recorded on a 4-point Likert scale, with scores ranging from 0 (not true at all) to 3 (true all the time), with higher scores indicating higher resilience. The Cronbach’s *α* coefficient of CD-RISC in bariatric surgery candidates and post-bariatric surgery patients was 0.94 and 0.96, respectively.

#### 2.2.3 Anxiety

The Chinese version of the Generalized Anxiety Disorder 7-item scale (GAD-7) was used to screen for anxiety symptoms (29). Participants were asked to rate how often have they been bothered by seven problems in the past two weeks, such as “feeling nervous, anxious, or on edge.” All items were rated on a 4-point Likert scale ranging from 0 (not at all) to 4 (nearly every day). The higher the score, the more severe was the anxiety. The Cronbach’s *α* coefficient of GAD-7 in bariatric surgery candidates and post-bariatric surgery patients was 0.89 and 0.85, respectively.

#### 2.2.4 Depression

The Patient Health Questionnaire Depression Scale (PHQ-9) is used to diagnose depression based on the DSM-IV depression disorder criteria (30). PHQ-9 assess individual’s depression by 9 depression symptoms (e.g., “Little interest or pleasure in doing things?”) which may bother them within two weeks. The items were 4-point Likert type, with responses ranging from 0 (not at all) to 3 (nearly every day). The Cronbach’s *α* coefficient of PHQ-9 in bariatric surgery candidates and post-bariatric surgery patients was 0.87 and 0.75, respectively.

### 2.3 Statistical analysis

Descriptive statistics and correlations between variables among bariatric surgery candidates and post-bariatric surgery patients were analyzed using SPSS24.0. The differences in mental health between bariatric surgery candidates and post-bariatric surgery patients were examined through an independent sample t-test in R. The common method bias, discriminant validity analysis, and mediation model analysis for bariatric surgery candidates and post-bariatric surgery patients were conducted using Mplus8.3, respectively.

## 3 Results

### 3.1 Discriminant validity

A four-factor model and three alternative models were constructed using confirmatory factor analysis (CFAs). The results showed that the four-factor models performed better than the other three models in both bariatric surgery candidates and post-bariatric surgery patients (see **Table 1**), so insomnia resilience, anxiety, and depression were distinct from each other.

**Table 1:** Results of four models constructed by confirmatory factor analyses.

Group	Model	Factors	*χ^2^/df*	*CFI*	*TLI*	*RMSEA*	*SRMR*
BSC group	Four-factor	F1, F2, F3 F4	2.54	0.858	0.851	0.060	0.063
	Three-factor	F1+F2, F3, F4	2.69	0.843	0.836	0.063	0.065
	Two-factor	F1+F2+F3, F4	3.53	0.765	0.754	0.077	0.080
	One-factor	F1+F2+F3+F4	6.32	0.506	0.484	0.111	0.149
post-BS group	Four-factor	F1, F2, F3 F4	1.82	0.767	0.756	0.083	0.088
	Three-factor	F1+F2, F3, F4	1.82	0.767	0.756	0.083	0.090
	Two-factor	F1+F2+F3, F4	2.10	0.686	0.672	0.096	0.104
	One-factor	F1+F2+F3+F4	2.74	0.504	0.482	0.121	0.148

BSC group, bariatric surgery candidate group; post-BS group, post bariatric surgery patient group.

F1, anxiety; F2, depression, F3, insomnia; F4, resilience.

### 3.2 Common method bias

The Harman single-factor test was conducted to test for common method bias in both the bariatric surgery candidates and post-bariatric surgery patients. The first factor explained 29.00% of the total variance in bariatric surgery candidates, with a rate of 30.91% in post-bariatric surgery patients.

We also examined common method bias using the unmeasured latent method factor (31). Compared with the four-factor model, the increase in TLI of unmeasured latent methods factor in both bariatric surgery candidates and post bariatric surgery patients were less than 0.05 (BSC: *χ^2^/df* = 2.32, CFI = 0.884, TLI = 0.872, RMSEA = 0.055, SRMR = 0.051; post-BS: *χ^2^/df* = 1.69, CFI = 0.814, TLI = 0.796, RMSEA = 0.076, SRMR = 0.069), satisfy contemporary criteria for excluding substantial common method bias (32, 33).

### 3.3 Preliminary analysis

The descriptive statistics and correlations of all variables are presented in **Table 2**. There were significant correlations between all variables among bariatric surgery candidates and post-bariatric surgery patients, except for resilience and insomnia. In addition, the results of the independent sample t-test showed that the levels of insomnia, anxiety, and depression decreased significantly after bariatric surgery (**Table 2**).

**Table 2:** Descriptives statistics and associations between variables of interest.

Variables	BSC group				Post-BC group				*t*
	M ± SD	1	2	3	M ± SD	1	2	3	
1.insomnia	6.08 ± 6.96	/			3.52 ± 4.67	/			5.62^***^
2.resilience	67.90 ± 16.40	0.03	/		65.56 ± 17.38	-0.08	/		0.26
3.anxiety	2.93 ± 3.14	0.50^**^	−0.22^**^	/	2.07 ± 2.68	0.46^**^	−0.27^**^	/	3.63^***^
4.depression	5.65 ± 4.92	0.75^**^	−0.16^**^	0.74^**^	4.10 ± 3.48	0.71^**^	−0.24^**^	0.73^**^	4.69^***^

BSC group, bariatric surgery candidate group; post-BS group, post bariatric surgery patient group.

*t*, the results of the independent samples t-test.

^**^ *p* <.01; ^***^ *p* <.001.

### 3.4 Mediating role of resilience and anxiety

We first examined the chain-mediating effect of resilience and anxiety on the relationship between insomnia and depression among bariatric surgery candidates (**Table 3**). The results showed that insomnia had a significant direct effect of on depression. Resilience functioned as a mediator in the relationship between insomnia and depression as well as anxiety. The results also indicated that resilience and anxiety play a chain-mediating role in the relationship between insomnia and depression.

**Table 3:** The results of mediation effect analysis.

Group	Path	*β*	*95%CI*	*p*	Indirect effect
BSC group	insomnia→depression	0.492	(0.419,0.568)	<0.001	/
	insomnia→resilience→depression	0.023	(0.009,0.042)	<0.01	3.07%
	insomnia→anxiety→depression	0.209	(0.164,0.304)	<0.001	27.90%
	insomnia→resilience→anxiety→depression	0.024	(0.012,0.042)	<0.01	3.20%
post-BS group	insomnia→depression	0.481	(0.323,0.602)	<0.001	/
	insomnia→resilience→depression	0.002	(−0.006,0.028)	ns	/
	insomnia→anxiety→depression	0.281	(0.177,0.411)	<0.001	36.64%
	insomnia→resilience→anxiety→depression	0.004	(−0.012,0.028)	ns	/

Standardized coefficients are reported.

BSC group, bariatric surgery candidate group; post-BS group, post bariatric surgery patient group.

ns, not significant.

We then tested the mediating effects of resilience and anxiety on the relationship between insomnia and depression among post-bariatric surgery patients (**Table 3**). The results showed that insomnia was significantly positively correlated with depression. However, only anxiety plays a mediating role in the relationship between insomnia and depression.

## 4 Discussion

Our study explored the psychological mechanisms underlying the relationship between insomnia and depression in bariatric surgery candidates and post-bariatric surgery patients. For bariatric surgery candidates, the results revealed that insomnia was positively related to depression. Resilience, anxiety and the chain from resilience to anxiety mediated the relationship between insomnia and depression. However, in post-bariatric surgery patients, only a direct effect of insomnia on depression and a mediating role of anxiety were observed.

Significant mental health improvements were observed among post-bariatric surgery patients, consistent with prior research documenting the psychological benefits of bariatric surgery. Specifically, studies have shown significant reductions in depression and anxiety symptoms (34, 35) along with noticeable improvements in sleep quality and duration after bariatric surgery (24, 36). These psychological benefits may stem from post-bariatric improvements in body image perception, subsequently enhancing mental health outcomes (37).

Accumulating evidence has established insomnia as a predictor of depression across diverse populations (38–40). Similarly, our study found that insomnia was significantly positively related to depression in both bariatric surgery candidates and post-bariatric surgery patients. This consistency across samples may be attributable to the impairment of emotion regulation processes caused by chronic sleep deprivation, potentially through lasting alterations in limbic system reactivity and prefrontal cortical function (41), which are not fully normalized by metabolic improvements alone.

Additionally, anxiety mediates the relationship between insomnia and depression in both bariatric surgery candidates and post-bariatric surgery patients. Although previous research has not directly explored the relationship between insomnia, anxiety, and depression, studies have shown that insomnia can lead to negative outcomes through the sequential mediation of anxiety and depression (42, 43). As previously mentioned, insomnia may hinder individuals’ capacity to manage negative emotions, leading to elevated levels of anxiety (41), which in turn amplifies depressive symptoms (19).

Interestingly, the mediating role of resilience and the chain mediating effect of resilience and anxiety were observed only among bariatric surgery candidates. It appears that resilience functions as a buffer in the present study. Stress and coping theory points out that when individuals perceive a situation as stressful, they evaluate the available coping resources (44, 45). Bariatric candidates often experience poor sleep, which may activate resilience as a protective factor. However, after bariatric surgery, sleep quality improves significantly and insomnia may no longer be perceived as a salient stressor. Consequently, resilience may no longer be activated.

## 5 Implications and limitations

First, the underlying psychological mechanisms linking insomnia to depression in bariatric surgery patients were identified. Second, the differences in the mediating role of resilience between bariatric surgery candidates and post-bariatric surgery patients provide evidence that resilience may be more salient in high-risk environments. Clinically, the unique mediating role of resilience in bariatric surgery candidates underscores the necessity of implementing targeted interventions to enhance coping capacity against severe insomnia. Additionally, patients’ insomnia symptoms warrant continued attention, even after the completion of bariatric surgery.

Our investigation was exploratory, and some limitations were applied. First, its cross-sectional design limited its ability to establish causal relationships between insomnia and depression. Moreover, while resilience showed differential mediation effects in bariatric surgery candidates and post-bariatric surgery patients, it did not provide insight into the trajectory of these changes over time. Third, although the current study examined insomnia, resilience, and anxiety as key factors in developing depression, future research should integrate additional psychosocial determinants (e.g., social support and socioeconomic status) to account for depression’s multifactorial nature. Fourth, while the one-month follow-up offers valuable preliminary data on short-term psychological adaptation, this duration may not adequately reflect the complete developmental course of resilience and mental health changes in bariatric surgery patients. Finally, previous studies have indicated potential bidirectional relationships among insomnia, anxiety, and depression. In future studies, further investigation of the dynamic interactions among these variables should be conducted.

## 6 Conclusion

This study is of great significance in improving the mental health of bariatric surgery patients. It has been suggested that improving bariatric surgery patients’ insomnia and reducing anxiety levels can help decrease their depression levels. In bariatric surgery candidates, resilience plays a greater protective role in the impact of insomnia on depression.

## Data Availability

The raw data supporting the conclusions of this article will be made available by the authors, without undue reservation.
